# Genetic landscape of Gullah African Americans

**DOI:** 10.1002/ajpa.24333

**Published:** 2021-05-19

**Authors:** Kip D. Zimmerman, Theodore G. Schurr, Wei‐Min Chen, Uma Nayak, Josyf C. Mychaleckyj, Queen Quet, Lee H. Moultrie, Jasmin Divers, Keith L. Keene, Diane L. Kamen, Gary S. Gilkeson, Kelly J. Hunt, Ida J. Spruill, Jyotika K. Fernandes, Melinda C. Aldrich, David Reich, W. Timothy Garvey, Carl D. Langefeld, Michèle M. Sale, Paula S. Ramos

**Affiliations:** ^1^ Center for Precision Medicine Wake Forest School of Medicine Winston‐Salem North Carolina USA; ^2^ Department of Anthropology University of Pennsylvania Philadelphia Pennsylvania USA; ^3^ Center for Public Health Genomics University of Virginia Charlottesville Virginia USA; ^4^ Department of Public Health Sciences University of Virginia Charlottesville Virginia USA; ^5^ Gullah/Geechee Nation St. Helena Island South Carolina USA; ^6^ Lee H. Moultrie & Associates North Charleston South Carolina USA; ^7^ Department of Health Services Research New York University Winthrop Hospital Mineola New York USA; ^8^ Department of Biology East Carolina University Greenville North Carolina USA; ^9^ Center for Health Disparities East Carolina University Brody School of Medicine Greenville North Carolina USA; ^10^ Department of Medicine Medical University of South Carolina Charleston South Carolina USA; ^11^ Department of Public Health Sciences Medical University of South Carolina Charleston South Carolina USA; ^12^ College of Nursing Medical University of South Carolina Charleston South Carolina USA; ^13^ Department of Thoracic Surgery Vanderbilt University Medical Center Nashville Tennessee USA; ^14^ Department of Medicine Vanderbilt University Medical Center Nashville Tennessee USA; ^15^ Department of Biomedical Informatics Vanderbilt University Medical Center Nashville Tennessee USA; ^16^ Vanderbilt Genetics Institute Vanderbilt University Medical Center Nashville Tennessee USA; ^17^ Department of Genetics Harvard Medical School Boston Massachusetts USA; ^18^ Howard Hughes Medical Institute Harvard Medical School Boston Massachusetts USA; ^19^ Broad Institute of MIT and Harvard Cambridge Massachusetts USA; ^20^ Department of Human Evolutionary Biology Harvard University Cambridge Massachusetts USA; ^21^ Department of Nutrition Science University of Alabama at Birmingham Birmingham Alabama USA

**Keywords:** admixture, ancestry, demography, slavery, West Africa

## Abstract

**Objectives:**

Gullah African Americans are descendants of formerly enslaved Africans living in the Sea Islands along the coast of the southeastern U.S., from North Carolina to Florida. Their relatively high numbers and geographic isolation were conducive to the development and preservation of a unique culture that retains deep African features. Although historical evidence supports a West‐Central African ancestry for the Gullah, linguistic and cultural evidence of a connection to Sierra Leone has led to the suggestion of this country/region as their ancestral home. This study sought to elucidate the genetic structure and ancestry of the Gullah.

**Materials and Methods:**

We leveraged whole‐genome genotype data from Gullah, African Americans from Jackson, Mississippi, African populations from Sierra Leone, and population reference panels from Africa and Europe to infer population structure, ancestry proportions, and global estimates of admixture.

**Results:**

Relative to non‐Gullah African Americans from the Southeast US, the Gullah exhibited higher mean African ancestry, lower European admixture, a similarly small Native American contribution, and increased male‐biased European admixture. A slightly tighter bottleneck in the Gullah 13 generations ago suggests a largely shared demographic history with non‐Gullah African Americans. Despite a slightly higher relatedness to populations from Sierra Leone, our data demonstrate that the Gullah are genetically related to many West African populations.

**Discussion:**

This study confirms that subtle differences in African American population structure exist at finer regional levels. Such observations can help to inform medical genetics research in African Americans, and guide the interpretation of genetic data used by African Americans seeking to explore ancestral identities.

## 1 INTRODUCTION

Knowledge about the genetic background of a population, including regional differences in ancestry, structure, and variation, is not only critical for medical and population genetic studies (Moreno‐Estrada et al., 2014), but can also illuminate questions of social and cultural relevance. Multiple studies show wide variability in the levels of African ancestry in African American individuals in the United States (U.S.) at both state and regional levels (Baharian et al., 2016; Bryc, Durand, Macpherson, Reich, & Mountain, 2015; Dai et al., 2020; Han et al., 2017; Mathias et al., 2016; Micheletti et al., 2020; Patin et al., 2017). This variation in African ancestry underscores the importance of properly accounting for ancestry in historical and biomedical studies of diasporic populations (Reiner et al., 2005; Shriner, Tekola‐Ayele, Adeyemo, & Rotimi, 2014).

While regional patterns in ancestry proportions in African Americans in the U.S. are broadly understood, fine‐scale characterization of the ancestral diversity of discrete groups is lacking. In addition, as a result of the trans‐Atlantic slave trade, African Americans were robbed of their African heritage and left with limited information about ancestors and homelands (Rotimi, 2003). The longing for identity and belonging leads many African Americans to actively draw together and evaluate various sources of genealogical information (historical, social and genetic) in order to weave together ancestry narratives (Nelson, 2008). Elucidating the African ancestries of African Americans can enrich the lives of African Americans by helping them to enrich a sense of identity, make connections to ancestral homelands, and ultimately foster reconciliation in the wake of emancipation (Nelson, 2016).

The Gullah are a culturally distinctive group of African Americans from the coastal Sea Islands of North Carolina, South Carolina, Georgia, and Florida. On many plantations of the coastal Sea Islands, Africans vastly outnumbered Europeans. Their relative isolation fostered the development of a unique culture in which many African influences were preserved, including language, folktales, religious beliefs, food preferences, music, dance, arts, and crafts (Jackson et al., 2005; Parra et al., 1998).

Marked by unique intonation and rhythm as well as syntax and lexicon, the Gullah language is hardly intelligible to the outsider, and remains the most characteristic feature of the sea islanders. The origin of this unique Creole language, like that of the Gullah people, is still debated. One hypothesis proposes that they descend from Krio ancestors originating in Sierra Leone (Hancock, 1969; Opala, 1987), a view supported by the fact that contemporary sea islanders can understand the Krio of Sierra Leone and vice versa. This striking linguistic resemblance, coupled with multiple cultural links (e.g., rice growing techniques, quilts, songs, stories), has led to a commonly held narrative that the Gullah are descendants of enslaved Africans from the African Rice Coast (Opala, 1987), the traditional rice‐growing region which stretches south from Senegal to Sierra Leone and Liberia (Figure S1).

Other historical accounts support a diverse African ancestry of the Gullah (Brady, 1972; Nash, 2010; Pollitzer, 1999). The recorded legal slave trade into Charleston, South Carolina, documents approximately 39% of enslaved Africans as originating from West‐Central Africa (present day Angola, Congo, and part of Gabon), 20% from Senegambia (present day Senegal and Gambia), 17% from the Windward Coast (present day Ivory Coast and Liberia), 13% from the Gold Coast (present day Ghana), 6% from Sierra Leone (present day Sierra Leone and Guinea), and 5% from the Bights of Benin and Biafra (Togo, Benin, Nigeria, Cameroon, and part of Gabon) (Figure S1) (Pollitzer, 1999). In addition to cultural links (e.g., religious beliefs, arts and crafts), words and syntax support a larger role of West‐Central Africa, the Gold Coast, and adjacent Nigeria in forming the Gullah language (Cassidy, 1980). While it has been proposed that the Congo‐Angola area had an early cultural dominance with artifacts, lexicon and beliefs, the complexities of the Bantu grammar probably prevented its adoption in the Sea Islands. Senegambia, Sierra Leone, and the Windward Coast down through the Bight of Biafra contributed most in the latter half of the 18th century, when half of all slaves imported into Charleston arrived, adding more words, grammar, and even whole stories (Pollitzer, 1999). Thus, it is likely that, instead of Gullah deriving directly from Krio, both languages share a close common origin (Pollitzer, 1999).

In this context, it is currently unknown how significant of a genetic legacy that Sierra Leone ancestors might have left in present‐day Gullah African Americans. Early genetic studies of autosomal, mtDNA, and Y‐chromosome markers, indicated that the Gullah had high African ancestry (Parra et al., 1998; Parra et al., 2001) and a lower genetic distance to populations from Sierra Leone compared with African Americans from urban areas (McLean Jr. et al., 2005; McLean Jr. et al., 2003). Sierra Leone is also officially home to sixteen different ethnic groups, each with its own language and customs. The largest native ethnic groups include the Temne (35%) in northern Sierra Leone and areas around the capital, who arrived during the 11th and 12th centuries upon the fall of the Jalunkandu Empire in present day Republic of Guinea (Bangura, 2017; Rodney, 1970; Taylor, 2014; Wylie, 1977). The Mende (31%), who live mostly in the Southeast and the Kono District, appear to have early origins from people of the Empires of the Western Sudan (near present‐day Mali), who migrated from the inland to the coast between the 2nd and 16th centuries to trade woven cloths for salt (Abraham, 2003; Fage, 2001), as well as more recent contributions from the Mane invasions of the 16th century (Dwyer, 2005; Ogot, 1992). The Limba (8%) are native to the savannah‐woodland region in northern Sierra Leone, with many having moved to Freetown to escape capture by slave traders and transportation to North America (Alie, 1990; Finnegan, 1965). By contrast, the Fula (7%) are descendants of Fulani migrant from Guinea who settled in Sierra Leone during the 17th and 18th centuries (Fyfe, 1979; Knörr & Kohl, 2016). Likewise, the Kono (5%) and the Mandingo (2%) are descendants of Guinea migrants (Knörr & Kohl, 2016) (Figure S2).

The Creole (2%) are descendants of freed African slaves from America who settled in Sierra Leone after 1787, and as such have multiple African origins (Fyfe, 1979). Following the American Revolutionary War (1775–1783), the British government freed Africans who served in the British armed forces and resettled them in Granville Town, the predecessor of Freetown and the present capital of Sierra Leone. Maroons, runaway enslaved Africans from the West Indies who formed independent settlements on different islands, were also resettled in Freetown, as were over 50,000 “recaptives” brought there by the British navy (Cole, 2013). The subsequent generations born in Sierra Leone were called Krio, Kriole, or Creole.

Individuals from other Sierra Leone ethnic groups joined the Creole communities, thereby promoting a fusion of African and Western cultures (Dixon‐Fyle & Cole, 2006). The Krio language unites the different ethnic groups for trade and interactions with each other (Oyètádé & Fashole‐Luke, 2008). The present national boundary was only fixed in 1896, prior to which people moved freely through the coastal country, making their own settlements, and fixing their own boundaries between themselves and their neighbors (Fyfe, 1979).

This study sought to elucidate the population structure of the Gullah and their relationship to contemporary Sierra Leone ethnic groups and other West African populations using genome‐wide genotype data. African Americans from the Jackson Heart Study (JHS) (Musunuru et al., 2010; Taylor Jr. et al., 2005) were also included to provide a comparison with a less geographically isolated, but regionally close, Southeastern U.S. African American sample. The results of this analysis support the complex African ancestry and reduced European admixture of the Gullah compared to other U.S. African American populations. These results are consistent with historical data (Pollitzer, 1999), which indicate that the Gullah are a mixture of numerous people from different genetic, ethnic, and linguistic currents who formed their own culture and language. Identifying the diverse people who played a role in shaping the Gullah has implications for all African Americans, and for the legacy of the African diaspora everywhere (Pollitzer, 1999).

## 2 SUBJECTS AND METHODS

### 2.1 Community engagement

This study was conducted in cooperation with and approval from the Sea Island Families Project (SIFP) Citizen Advisory Committee (Spruill et al., 2013). Interdisciplinary research teams from the Medical University of South Carolina (MUSC) developed community‐engaged research projects between the academic researchers and Gullah African Americans residing in rural South Carolina, leading to the formation of SIFP Citizen Advisory Committee (Spruill et al., 2013). This partnership has been ongoing for over 20 years. Participants from three SIFP projects were included in our study: the Sea Island Genetic African American Registry (Project SuGAR) (Divers et al., 2010); the Center of Biomedical Research Excellence (COBRE) for Oral Health pilot project, “An Epidemiological Study of Periodontal Disease and Diabetes: Cytokine Genes and Inflammation Factors” (Fernandes et al., 2009); and the Systemic Lupus Erythematosus in Gullah Health (SLEIGH) Study (Kamen et al., 2008). The SIFP Citizen Advisory Committee meets quarterly for sharing of research results and providing guidance and recommendations to new research. The SIFP Citizen Advisory Committee agreed to this study aimed at understanding the population genetics and ancestry of the Gullah.

### 2.2 Sample collection and SNP data generation

Self‐identified Gullah African American subjects in our study from the aforementioned SuGAR (Divers et al., 2010), COBRE for Oral Health (Fernandes et al., 2009), and SLEIGH (Kamen et al., 2008) projects were recruited under ongoing protocols approved by the MUSC Institutional Review Board and adhered to the tenets of the Declaration of Helsinki. All self‐identified Gullah African American participants and their parents were born and raised in the Sea Islands region of South Carolina, or South Carolina low country (along the coastal border and 30 miles inland). The vast majority of Gullah participants was recruited through scheduled community health fairs, recruitment events at local churches, medical clinics, and established organizations on the Sea Islands (Divers et al., 2010; Sale et al., 2009). The major sites of recruitment spanned the Charleston and Beaufort counties, including Saint James‐Santee, Johns Island, Edisto Island, Saint Helena Island, and Daufuskie Island.

All subjects received a general medical examination, donated blood samples for DNA analysis, and provided basic demographic and ethnic information. DNA was extracted from blood using a standardized DNA isolation kit (Gentra Systems, Minneapolis, MN). Sample collection and processing have been previously described for the African American subjects from the Jackson Heart Study (JHS) (Musunuru et al., 2010; Taylor Jr. et al., 2005), Native American Mixtec (Raghavan et al., 2015), and Sierra Leonean (Jackson et al., 2005) subjects. We note that, while Gullah African Americans represent a predominantly rural sample, JHS participants represent the urban, metropolitan area of Jackson, Mississippi. Of note, while the Creole from Sierra Leone were largely recruited in Freetown, and are thus a more urban group, the other ethnic groups were recruited in smaller towns and communities where ethnic communities had lived for many years and the ethnic affiliations were distinct and reliable, and are thus representative of more rural areas. In summary, demographic representativeness was attempted during sampling.

DNAs from the Gullah and Sierra Leone African subjects were genotyped using the Affymetrix Genome‐Wide Human SNP Array 6.0 at the Children's Hospital of Philadelphia's (CHOP) Center for Applied Genomics. The Bayesian robust linear model with Mahalanobis (BRLMM‐P) algorithm was used to generate SNP calls, and additional quality control (QC) was performed to exclude SNPs with low genotype call rates (<95%), low minor allele frequency (MAF < 0.05), and genotypes inconsistent with Hardy Weinberg Equilibrium (HWE) (*P* < 10^−10^). Samples were excluded for low call rate (<95%) and for gender inconsistencies between recorded and genotype‐inferred sex. Duplicates, first‐, second‐, and third‐degree relatives were also excluded, with unrelatedness being defined as pair‐wise kinship coefficients smaller than 0.0442 estimated by Kinship‐based Inference for GWAS (KING) software v2.1.2 (Manichaikul et al., 2010).

**FIGURE 1:**
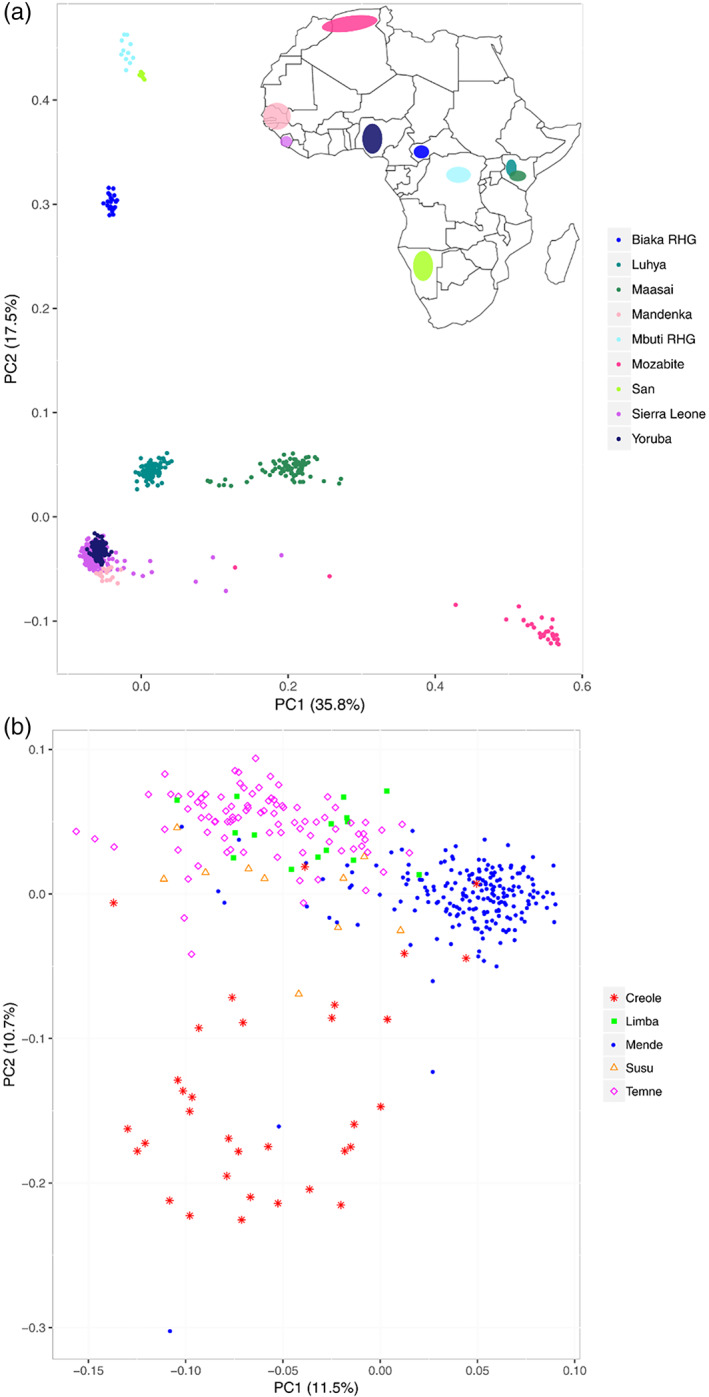
Principal component analysis of all African samples. Principal component analysis (PCA) (EIGENSOFT) was applied to HGDP and HapMap III African and Sierra Leonean populations. (a) PCA showing the Mozabite cluster (along PC1), the West, Eastern and Southern, and Middle and South Western subpopulations clusters (along PC2). Insert shows approximate locations of sampled populations in Africa. (b) PCA of Sierra Leone ethnic groups with *n* > 10 showing the Mende, Creole and Temne forming relatively different, but overlapping, clusters

Prior to QC procedures, 1558 Gullah African Americans, 1775 JHS African Americans, 400 Sierra Leone Africans, and 8 Mixtec individuals were available for our analysis. The Mexican Mixtec individuals have >99% Native American ancestry estimated by ADMIXTURE (Alexander, Novembre, & Lange, 2009). After QC procedures, 883 unrelated Gullah African Americans, 1322 unrelated JHS African Americans, 381 unrelated Sierra Leone Africans, and 7 Mixtec subjects were retained for analyses (Table S1). In addition, 125 unrelated Stanford‐Human Genome Diversity Project (HGDP) (Li et al., 2008) and 386 unrelated HapMap III (release 3) (Altshuler et al., 2010) subjects were used for analysis. The geographic and linguistic distribution of the African populations used in this study are shown in Table S2 and Figure 1a.

### 2.3 Data merging and SNP trimming

PLINK v1.9 (Chang et al., 2015) was used to combine our Affymetrix 6.0 data, HapMap III release 3 data, and HGDP data. After merging samples, 136,878 common variant SNPs were retained after removing SNPs (1) with strand problems, (2) occurring in the HLA region, (3) having a MAF < 0.01, (4) showing HWE < 1 × 10^−10^, and (5) having a missing rate > 0.05. For methods that required a set of linkage disequilibrium (LD)‐pruned SNPs (see below), we further removed SNPs with an *r* ^2^ > 0.1, leaving 64,303 uncorrelated SNPs for analysis. For more specific analyses, identical merging and filtering methods were used to combine the Affymetrix 6.0 data with the HapMap III data. This step resulted in a combined set of 27,374 pruned and filtered SNPs for the Gullah and 29,277 for the non‐Gullah (JHS) African Americans.

### 2.4 Principal component analysis for inference of population structure

Principal component analysis (PCA) as implemented in EIGENSOFT v6.0.1 (Patterson, Price, & Reich, 2006) was computed with the Sierra Leone African samples combined with all HGDP and HapMap III African samples, for African subpopulation structure inference. For African American population structure inference, HGDP and HapMap III European samples were added to the African samples as references. PCA analyses were performed using the set of LD‐pruned 64,303 SNPs described above. To test for a significant shift in principal components between the Gullah and JHS, a PerMANOVA was computed for the sets of principal components between PC1 and PC6 (6 PCs explain >90% of the total variance). In addition, Wilcoxon signed‐rank tests were computed on each individual principal component to test for differences between the Gullah and JHS.

### 2.5 Admixture inference

Unsupervised clustering as implemented in ADMIXTURE v1.3.0 (Alexander et al., 2009) was used to estimate global genetic ancestry of the European, African, and African‐American populations. ADMIXTURE analysis was performed in European, African (including Sierra Leonean populations), and African American individuals, assuming 2 through 8 ancestral genetic clusters (*k* = 2 through *k* = 8) to determine the optimal number of ancestral reference groups. Five clusters (i.e., *k* = 5) gave the lowest cross‐validation error (Figure S3). To help order the populations according to their genetic similarities, we used average linkage hierarchical cluster analysis based on the means of each of the five ancestral populations computed by ADMIXTURE v1.3.0 (Alexander et al., 2009) and inter‐population similarity matrix of Euclidean distances. The resulting dendrogram is shown in Figure S4.

ALDER v1.03 (Loh et al., 2013) was used to infer admixture timing in Gullah and JHS African Americans on the basis of exponential decay of linkage disequilibrium. ALDER was run separately for the Gullah and JHS using the YRI and CEU as reference populations. ALDER was computed with a minimum number of four individuals from the test population needing successful genotype calls at a SNP in order for the SNP to be used. The bin size used was 0.0005 cM and the minimum genetic distances at which to start and stop curve fitting were specified to 0.1 cM and 0.5 cM, respectively.

### 2.6 Inference of ancestry proportions

To generate estimates of ancestry on the autosomes and chromosome X for both the Gullah and JHS African‐Americans, we ran qpAdm (Haak et al., 2015). This program leverages allele frequency correlations between the admixed and source populations with distant outgroups to eliminate potential biases due to genetic drift between the true source populations and the ones used as surrogates for them. The HapMap CEU, HapMap YRI, and Mixtec (kindly provided by Drs. Raghavan and Willerslev (Raghavan et al., 2015)) were used as source reference populations for European, African, and Native American ancestry, respectively. It is important to note that, unlike other admixed Native Americans, the Mexican Mixtec individuals (Raghavan et al., 2015) have >99% Native American ancestry as estimated by ADMIXTURE (Alexander et al., 2009). For the outgroups, the Luhya (LWK), Maasai (MKK), Han Chinese (CHB), Japanese (JPT), Gujarati Indians (GIH), and Toscani (TSI) populations from HapMap were used.

qpAdm (Haak et al., 2015) was run on 798 females from the JHS cohort and 680 females from the Gullah cohort. A total of 523,638 SNPs were used to estimate ancestry on the autosomes and 20,879 SNPs were used to estimate ancestry from the X‐chromosome. To determine sex‐biased admixture in the African American populations, we examined the African, European and Native American ancestry proportions between the X‐chromosomes and the autosomes in both the Gullah and the JHS African Americans for equality. To aid with interpretation, the proportion of ancestry that came from males was estimated under a simple model where, in a population with equally many females and males, the mean X‐chromosomal admixture fraction is a linear combination of female and male admixture parameters, with coefficients 2/3 and 1/3, respectively.

### 2.7 Detection of genomic segments shared identical‐by‐descent (IBD)

We used GERMLINE v1.5.1 (Gusev et al., 2009) to infer IBD tracts of length 18 cM or longer shared between Gullah and JHS African American individuals. GERMLINE was run under all of the default parameters with the exception of the minimum match length (−min_m) of 18 cM, treating each individual as two distinct and separate haplotypes (−haploid), 32 bits (−bits), and extending the match beyond the slice end to the first mismatching marker (−w_extend). Long IBD segments (*l* ≥ 18 cM) are informative of recent relatedness. Segments longer than 5 cM identified by GERMLINE have a negligible number of false positives (Durand, Eriksson, & McLean, 2014). IBD estimation was performed using the set of LD‐pruned 64,303 SNPs described above.

### 2.8 Estimation of effective population sizes

IBDNe (Browning et al., 2018) was implemented to estimate ancestry‐specific effective population size. The analysis excluded IBD segments with a “mincm” length shorter than 6, included 80 bootstrap samples, and set the maximum number of generations to estimate at 100. Ancestry‐specific IBDNe (ASIBDNe) was implemented following the exact sequence of steps for running ASIBDNe outlined by the creators of the IBDNe software online (Browning et al., 2018). The analysis pipeline can be found at http://faculty.washington.edu/sguy/asibdne/. Briefly, data were phased and IBD segments were detected with Beagle (Browning & Browning, 2007) before applying RFMix (Maples, Gravel, Kenny, & Bustamante, 2013) to estimate ancestry by chromosomal segments. Once ancestry‐specific IBD segments were estimated, IBDNe was applied to each set of ancestry‐specific IBD segments.

### 2.9 Genetic diversity and population differentiation

To assess the amount of genetic variation within the Gullah and Sierra Leone populations, heterozygosity (HET) and inbreeding coefficients (*F*) were calculated using genotypic data on 273 healthy Gullahs and 381 Sierra Leoneans. After pruning SNPs in high LD (*r* ^2^ > 0.5) with PLINK v1.9 (Chang et al., 2015), the remaining 395 K shared SNPs between these populations were used to calculate the HET and *F* statistics. For each individual, HET and *F* were estimated based on their expected and observed heterozygous calls, with *F* = (HETexp − HETobs)/(HETexp). The mean HET and mean *F* were calculated for each population and a two‐sample Wilcoxon test was used to test for a difference in HET and *F* between populations.

We computed the fixation index (*F*_ST_) (Weir & Cockerham, 1984) to inform about the genetic divergence between African Americans (Gullah and JHS) and their African and European ancestral populations. Using genotype data from 273 healthy Gullah, JHS African Americans, Sierra Leone, YRI and CEU samples, we computed the Weir and Cockerham's (1984) *F*_ST_ (Weir & Cockerham, 1984) as implemented in VCFtools v0.1.13 (Danecek et al., 2011). A total of 479 K autosomal SNPs were used for this analysis.

## 3 RESULTS AND DISCUSSION

### 3.1 Genetic structure of Sierra Leone Africans

**FIGURE 2:**
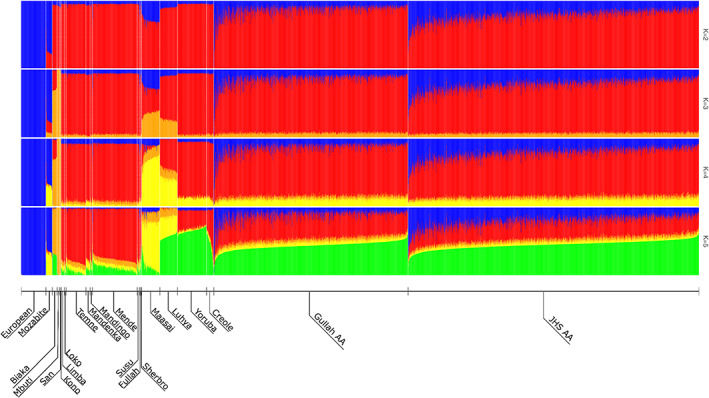
Ancestry estimates for European, African, and African American populations. ADMIXTURE analysis in Europeans, Africans (including Sierra Leone ethnic groups) and African Americans, assuming two through five ancestral genetic clusters (*k* = 2 through *k* = 5). The *k* = 5 setting has the lowest cross‐validation error of *k* = 2–8. Populations were ordered via hierarchical cluster analysis. The plot shows each individual as a thin vertical column colored in proportion to their estimated ancestry from one particular population. The initial distinction is between Europeans (blue) and Africans (other colors). Within Africans, red indicates a West African (aka African Rice Coast) ancestry (highest in the Mandenka of Senegal and Sierra Leone ethnic groups), orange a Central and South African ancestry (highest in the San of Namibia and Mbuti of Democratic Republic of the *Congo*), yellow an East African ancestry (highest in Maasai and Luhya of Kenya), and green a West‐Central African ancestry (aka Bight of Benin) ancestry (highest in the Yoruba of Nigeria)

Given the popularized narrative that Sierra Leone was the ancestral source of most Gullah African Americans, we first sought to characterize the population structure of African ethnic groups from this region. We combined genotype data from Sierra Leone Africans with Africans from the HGDP and HapMap III studies (Table S1), and inferred patterns of population structure and individual ancestry by principal component analysis (PCA) (Figure 1) and ADMIXTURE (Alexander et al., 2009) (Figure 2). Consistent with previous reports (see the review by (Gomez, Hirbo, & Tishkoff, 2014), PCA distinguished geographic and linguistic African subpopulations, separating a combination of geographic groups and speakers of the four major language families (Afro‐Asiatic, Nilo‐Saharan, Niger‐Kordofanian, and Khoisan) (Table S2).

As shown in Figure 1a, the first principal component (PC1) differentiated the Mozabites of North Africa from all other African populations. A few Mozabite and Sierra Leone individuals formed a geographical gradient, reflecting different levels of African and West‐Eurasian‐related admixture (Figure S5) (Henn et al., 2012). A slight partitioning of the East African groups also occurred, with the Maasai (Kenya) being separated from the Luhya (Kenya). PC2 further separated four main groups, with West Africans forming one cluster and the East African groups clustering together (Luhya and Maasai). The Biaka rainforest hunter‐gatherers (Central African Republic) formed an individual cluster, whereas the Mbuti rainforest hunter‐gatherers (Democratic Republic of the *Congo*) and San (Namibia) formed a more distant cluster. The relationship between population structure and geographic and linguistic factors is supported by the results from the global ancestry estimates (Figure 2 and Figure S4). ADMIXTURE (Alexander et al., 2009) (Figure 2) showed the highest West African‐associated ancestry in the Mandenka of Senegal and Sierra Leone ethnic groups (red), the highest East African‐associated ancestry in the Maasai and Luhya (yellow), and the highest Central and South African‐associated ancestry in the Mbuti and San, respectively (*orange*).

PCA was applied to data from Sierra Leone ethnic groups to further discriminate any potential clusters of genetic variation within the region (Figure 1b). Our results showed that the most populous ethnic groups (Mende and Temne) form relatively different, but overlapping clusters. The Limba clustered among the Temne, which was unexpected given their distinct, unrelated language, and the results of a previous analysis of mtDNA genetic diversity purporting that the Limba could be distinguished from the Mende, Temne, and Loko groups (Jackson et al., 2005). The Limba are indigenous to Sierra Leone, and their dialects are largely unrelated to the other languages in the region.

**FIGURE 3:**
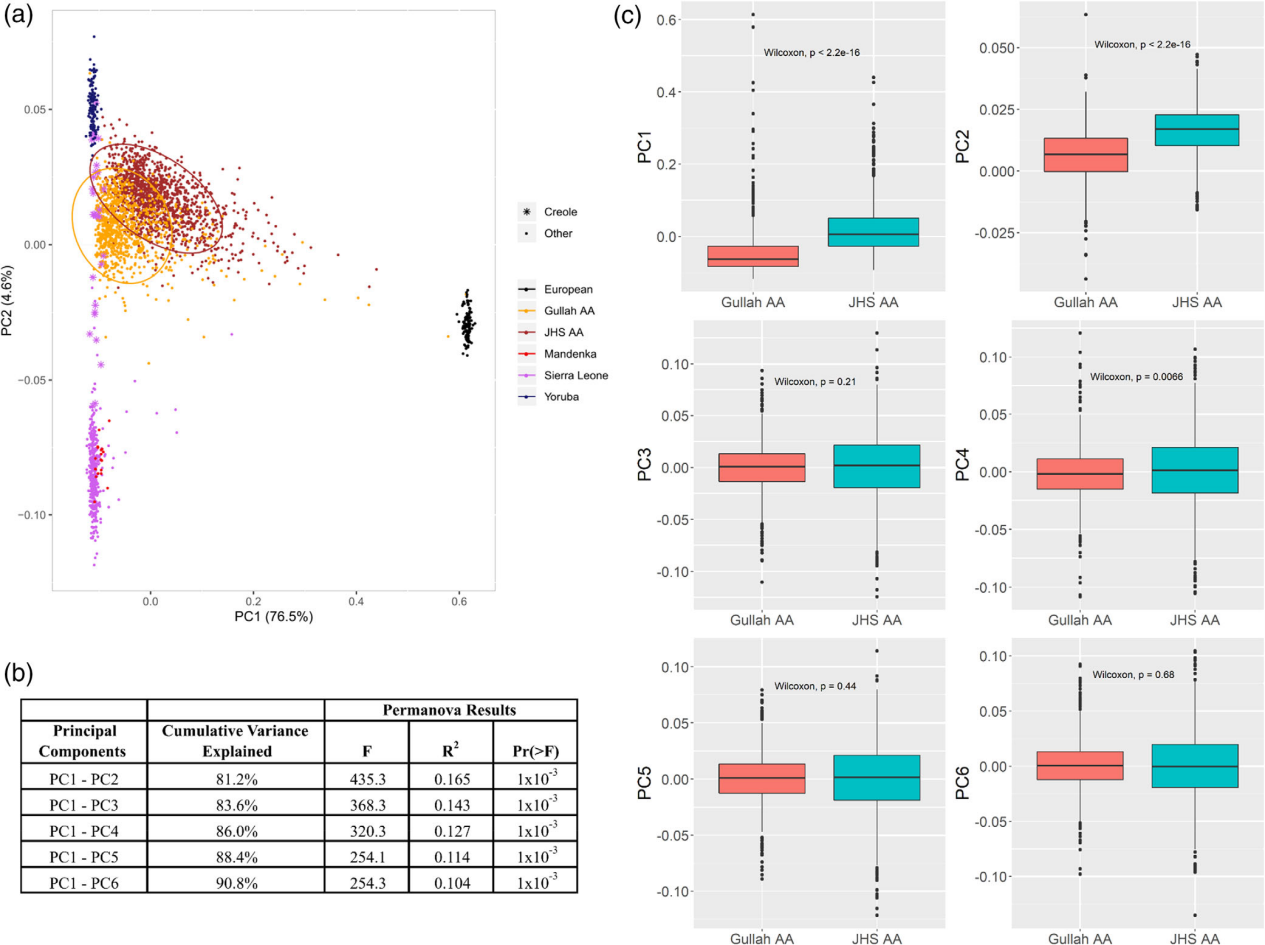
Principal component analysis of Gullah and non‐Gullah African Americans. (a) Principal component analysis (EIGENSOFT) using European (CEU samples from Utah) and African ancestral reference populations (YRI samples from Nigeria, Mandenka from Senegal, and Sierra Leone samples) illustrates the Gullah's closeness to Sierra Leone populations and the Mandenka, and also non‐Gullah (JHS) African Americans' proximity to the Yoruba. (b) To demonstrate the difference between Gullah and JHS African Americans, a PerMANOVA analysis was computed for each set of principal components between PC1 and PC6 to test for differences between the two groups; the resulting analyses each demonstrated significant differences (1 × 10^−3^), indicating a significant distance between the two groups. (c) Comparisons of each individual principal component between the Gullah and JHS. Wilcoxon p‐values are displayed. PC1 and PC2 appear to be the primary drivers in the differences between the Gullah and JHS. AA: African American; JHS: Jackson Heart Study African Americans from Jackson, Mississippi

The Krio or Creole formed a relatively distinct cluster along PC2. This pattern of population structure was broadly consistent with individual ancestry estimates (Figure 2 and Figure S4), whereas the Temne and Mende showed similar ancestry proportions, and the Creole appeared more variable in their African ancestry than other groups (Figure 2 and Figure S4). The Creole were also slightly more similar to the Yoruba, while other Sierra Leone ethnic groups showed more genetic similarity to the Mandenka (Figure 3 and Figure S4).

Interestingly, the Creole were genetically intermediate between other Sierra Leone ethnic groups and the Yoruba, with ancestral diversity similar to that of the Gullah African Americans. This finding suggested that they were likely the descendants of individuals originating from various parts of Africa, including Sierra Leone and beyond, as well as African descended individuals with European admixture. This finding is also consistent with their demographic history, which suggests the Creole descended from freed enslaved Africans (Fyfe, 1979) who mixed with other ethnic groups (Dixon‐Fyle & Cole, 2006). In summary, analysis of the African samples in this study showed that, despite the similarity among African Rice Coast populations (Mandenka and Sierra Leone ethnic groups), most of the recognized populations exhibit considerable genetic diversity.

### 3.2 African ancestry estimates of Gullah African Americans

To characterize the global patterns of ancestry and population structure of Gullah African Americans, we used qpAdm (Haak et al., 2015; Patterson et al., 2012), ADMIXTURE (Alexander et al., 2009), and PCA, with data from Gullah living in South Carolina, and for comparison with a regionally close group, from JHS African Americans. This analysis confirmed previous genetic reports of autosomal, mtDNA, and Y‐chromosome markers, i.e., that Gullah African Americans had lower European admixture and higher African ancestry than other African American populations in the USA (McLean Jr. et al., 2003; McLean Jr. et al., 2005; Parra et al., 1998; Parra et al., 2001). The higher average proportion of African ancestry in the Gullah was evident from autosomal global ancestry inference from qpAdm (Haak et al., 2015; Patterson et al., 2012), with the average African contribution to the Gullah African Americans being 90.7% compared with 82.2% in JHS African Americans (Table 1). Among studies of African ancestry in different U.S. regions (Baharian et al., 2016; Bryc et al., 2015; Dai et al., 2020; Mathias et al., 2016; Micheletti et al., 2020; Patin et al., 2017), the high African ancestry proportion seen in the Gullah was nearly matched in a U.S. cohort of African Americans sampled within rural Southeast U.S. (89% in Florida and 88% in South Carolina) (Baharian et al., 2016). Thus, the Gullah show the highest average African ancestry proportion of any U.S. African American group studied to date.

**TABLE 1:** Estimates of African, European, and Native American ancestry in two African American cohorts (% with 95% confidence intervals)

	Ancestry		
	African	European	Native American
**JHS African Americans (*n* = 1322)**			
Autosomes (*p*‐value = 0.00)	82.2% [82.1–82.3]	16.3% [16.2–16.4]	1.4% [1.3–1.5]
X‐chromosome (*p*‐value = 0.14)	86.5% [86.0–87.0]	11.8% [11.2–12.2]	1.7% [1.0–2.4]
Difference in mean X and autosomal ancestry	+4.3%	−4.5%	+0.3%
Estimated proportion of ancestry from males	42.15%	91.41%	17.86%
**Gullah African Americans (*n* = 883)**			
Autosomes (*p*‐value = 0.00)	90.7% [90.6–90.8]	8.0% [7.9–8.1]	1.3% [1.2–1.4]
X‐chromosome (*p*‐value = 0.26)	93.4% [92.9–93.9]	4.3% [3.7–4.9]	2.2% [1.1–3.1]
Difference in mean X and autosomal ancestry	+2.7%	−3.7%	+0.9%
Estimated proportion of ancestry from males	45.53%	119.38%	−53.85%

*Note*: Mean estimates (95% confidence intervals) of African, European, and Native American ancestry are shown. qpAdm was used to estimate proportions of European, African, and Native American ancestry, and qpAdm rank P‐values are listed for both cohorts for each of the autosomes and chromosome X analyses. The mean X‐chromosomal ancestry minus the mean autosomal ancestry (%) is listed. To aid with interpretation, the proportion of ancestry that comes from males was estimated under a simple model in which, in a population with equally many females and males, the mean X‐chromosomal admixture fraction is a linear combination of female and male admixture parameters, with coefficients 2/3 and 1/3, respectively. The proportion of European ancestry across all chromosomes is higher in the Jackson Heart Study (JHS) relative to the Gullah African Americans, but the proportion of European ancestry that comes from males is higher in the Gullah African Americans.

In parallel with the higher average African ancestry, the European ancestry estimate in the Gullah was the lowest reported for African Americans in the U.S., while that for the JHS African Americans was similar to estimates in other groups (e.g., as low as 14% and 15%) (Baharian et al., 2016; Bryc et al., 2015; Mathias et al., 2016) (Table 1). Consistent with the ancestry estimates, genome‐wide admixture analyses showed that, despite the highly variable levels of European and West African ancestry in all African American groups, the Gullah had a lower average level of European admixture than JHS African Americans (Figure 2). The lower European contribution in the Gullah corroborates known differences in ancestry proportions among African Americans in different U.S. states (Baharian et al., 2016; Bryc et al., 2015; Dai et al., 2020; Mathias et al., 2016; Micheletti et al., 2020; Patin et al., 2017), and confirms that subtle differences in African American population structure can exist at finer regional levels.

The lower European admixture in the Gullah was further corroborated by estimates of admixture timing in Gullah and JHS African Americans. To obtain a relative date of the admixture in the African American groups, we used admixture linkage disequilibrium (LD) decay, as implemented in ALDER (Loh et al., 2013). The weighted LD curve for the JHS African American group shows a steeper decay rate than that for the Gullah group as a function of genetic distance, suggesting earlier admixture in JHS African Americans (Figure S6). Accordingly, the admixture estimate for JHS African Americans dates to 4.5 generations ago, while for Gullah African Americans, the admixture estimate dates to 4.2 generations ago (Table 2). ALDER provides a relative date estimate for a single‐pulse admixture event, producing a single average date in cases of multiple pulses of admixture. Hence, these admixture estimates do not preclude earlier admixture events. Regardless, these estimates are close to the estimation of admixture time for HapMap African Americans in Southwest USA (ASW) of 5.4 generations ago using the approach by Zhou and colleagues (Zhou et al., 2017). In summary, the low European ancestry in the Gullah is supported by a more recent admixture date.

**TABLE 2:** Inference of admixture timing using ALDER

	Reference population						
Test population	A	B	*Z*‐score	Decay	Amplitude	Test status	Test *p*‐value
JHS AA	YRI	CEU	22.44	4.54 ± 0.18	3.4 × 10^−04^	Success	1.7 × 10^−111^
Gullah AA	YRI	CEU	25.15	4.21 ± 0.15	2.2 × 10^−04^	Success	1.3 × 10^−139^

*Note*: Decay shows the estimate for admixture date (in generations) and its block jackknifing standard error. JHS: Jackson Heart Study African Americans from Jackson, Mississippi; AA: African American; CEU: European reference samples from Utah; YRI: African reference samples from Nigeria.

The higher mean level of African ancestry in the Gullah is likely the result of the historically higher proportion of African Americans living in the Sea Islands of South Carolina since the early 1700s. African descendants comprised the majority of the population of South Carolina until the Great Migration to northern industrial cities in 1910 (Rogers & Taylor, 1994). The demand for enslaved Africans to work in the rice fields, and later in the cultivation of indigo and cotton, was very high through the 18th century and into the 19th century. As a result, there was a large influx of Africans into South Carolina and Georgia since the early colonial period (Parra et al., 2001). In the first federal census of 1790, enslaved Africans comprised 18% of the nation's total population, but ranged from 47–93% in several coastal areas of South Carolina, including the port of Charleston, the center of American slavery (Parra et al., 2001). During that time, while South Carolina had 43% enslaved Africans, the percentage for the Beaufort and Charleston Districts was much higher (76%) and that for the parish including the Sea Islands even higher (93%) (Fredrickson, 1981). Moreover, on the Sea Islands, all of the plantation owners who could leave the plantations from late May to late June would do so to avoid the risk of contracting malaria. The absence of planters during this period gave African descended individuals considerably more autonomy in developing their own culture there (Pollitzer, 1999).

Later, in 1860, when the enslaved African population in the US declined to 13%, that of South Carolina had risen to 57%. The approximately equal number of male and female slaves in these districts by 1810 suggests that the increase was due to reproduction of local populations rather than the importation of additional individuals from Africa. The increase in the number of Africans, their concentration in rural areas, the severity of slave codes, and the social alienation of Africans from Europeans, all contributed to the isolation of 18th century Gullah people. These conditions also provided an ideal context for creolization and the development of distinctive cultural attributes that continued into the 19th century and beyond (Pollitzer, 1999).

The higher mean level of African ancestry in the Gullah could be a direct effect of a potentially higher proportion of African Americans currently living in this region. Currently, in counties where the Gullah community members sampled for this study reside, African Americans encompass 20–50% of the population (United States Census Bureau, 2011). By contrast, the proportion of African Americans in the tri‐county area sampled for the Jackson Heart Study ranges from 20 to 70% (United States Census Bureau, 2011). The slightly higher proportion of African Americans in the Jackson tri‐county area suggests that the reported differences in population structure were not simply the result of current differential population proportions. Rather, the higher mean level of African ancestry in the Gullah reflects the historically higher proportion of African Americans living in the Sea Islands of South Carolina from the early 1700s through the mid‐1900s.

Finally, since African Americans living in rural areas have a higher average African ancestry than those living in urban areas (Baharian et al., 2016), we further considered the effects of sampling on ancestry proportions. The Gullah are an intrinsically rural community, while JHS participants represent urban dwellers. Baharian et al. (2016) report that, for both African Americans sampled only in rural, or in both urban and rural regions, the average African ancestry proportions are higher in South Carolina than in Mississippi. In support of this study, the proportion of African ancestry in JHS (82%) is similar to that reported by Baharian et al. (2016) in their urban and rural samples from Mississippi (83%), while the proportion of African ancestry in Gullah (91%) is slightly higher than that reported in their rural samples from South Carolina (88%) (Baharian et al., 2016). The 88% African admixture estimate in rural samples from South Carolina (Baharian et al., 2016) falls outside of the 95% confidence interval around the mean African ancestry of the Gullah ([90.6–90.8%]), suggesting that the African ancestry in the Gullah is significantly different than that for rural non‐Gullah African Americans in South Carolina. We thus infer that the slightly higher mean level of African ancestry in the Gullah might be an effect of their rural sampling, but also likely arose because of historical and sociocultural factors.

### 3.3 Native American ancestry estimates in Gullah African Americans

Consistent with early mtDNA and Y‐chromosome studies of Gullah African Americans (Parra et al., 2001), we found a small Native American contribution to the African American groups sampled for our study. We observed that the Gullah and JHS African Americans had slightly higher Native American ancestry than (~1.3–1.4%) had been reported in most African American groups in the U.S. (Baharian et al., 2016; Bryc et al., 2015; Dai et al., 2020; Mathias et al., 2016; Micheletti et al., 2020). This discrepancy may reflect the fact that previous studies have often used clustering methods like ADMIXTURE for estimating Native American ancestry proportions which are expected to give underestimates when the proxy population used for Native American ancestry (typically Mesoamerican) is highly genetically drifted from the true source population (Southeastern U.S. Native American). In contrast, the qpAdm ancestry estimation procedure explicitly accounts for genetic drift between the source population and the proxy population and produces an unbiased estimate. In the U.S., only African Americans living in the Southwest U.S. (ASW) from the 1000 Genomes Project had higher Native American ancestry (3.1%) (Martin et al., 2017).

This level of Native American ancestry is consistent with historical records about the Native American slave trade (Gallay, 2010; Pollitzer, 1999). In the early days of the American colonies, marriages were permitted between Europeans, Africans, and Native Americans (Pollitzer, 1999). Despite a 1671 law forbidding Native American slavery, Native Americans were publicly sold as slaves in Charleston, and their enslavement by colonists was common until the African slave trade accelerated in the 18th century. In fact, from 1670 to 1720, more Native Americans were shipped out of Charleston than Africans were imported (Gallay, 2010). After the Yamasee War (1715–17), Native American populations (Yamasee, Ochese, Waxhaw, Santee) declined in South Carolina, and most of the remaining Native American slaves were apparently absorbed into the African community (Pollitzer, 1999). The offspring of Africans and Native Americans were called *mustizoes* or *mustees*, in contrast to the *mulattoes* resulting from the union of Africans and Europeans.

From the 1730s through the 1780s, newspaper ads proclaimed that 2424 slaves had run away from their masters in South Carolina. As noted in 27% of the ads, a surprising 37% of runaways were described as being light, yellow, or mulatto in appearance, and 19% of them were said to be mustees (Pollitzer, 1999). The cultural influences of Native Americans are reflected in Gullah crafts, colono‐ware, boat building techniques, and decoctions of healing herbs used to cope with illness (Pollitzer, 1999). Further support for Native American admixture in the South comes from the several socially distinct communities with European, African, and American Indian ancestry that have persisted to the present day (e.g., Brass Ankles and Turks in South Carolina). In summary, historical, ethnographic, and mtDNA and Y‐chromosome data support a Native American contribution to the Gullah (Gallay, 2002; Gallay, 2010; Parra et al., 2001; Pollitzer, 1999), and this contribution is confirmed by our genome‐wide data.

### 3.4 Sex‐biased admixture in Gullah African Americans

To aid with interpretation, the proportion of ancestry that comes from males was estimated under a simple model in which, in a population with equally many females and males, the mean X‐chromosomal admixture fraction is a linear combination of female and male admixture parameters, with coefficients 2/3 and 1/3, respectively. We found evidence for patterns of sex‐biased gene flow in the Gullah (Table 1), consistent with the reported higher male European and female African contributions in other U.S. African Americans (Baharian et al., 2016; Bryc et al., 2015; Dai et al., 2020; Mathias et al., 2016; Micheletti et al., 2020; Patin et al., 2017), as well as previous work with the Gullah (Parra et al., 2001). The evidence for sex‐biased admixture is also in agreement with the smaller European paternal ancestry of African Americans in South Carolina relative to other North American and Caribbean groups reported in a large Y‐chromosome study (Torres, Doura, Keita, & Kittles, 2012).

The decrease in European ancestry on the X‐chromosome might imply a simultaneous European male bias and African female bias, which is consistent with increased frequency of sexual interactions between European males and African females, including rape and/or coerced sexual interactions (Kennedy, 2003; Lind et al., 2007). However, as shown by Goldberg and Rosenberg (2015), the difference in X chromosomal and autosomal admixture might also be the result of male biases in both Europeans and Africans. This interpretation would be consistent with the aforementioned asymmetric mating practices involving African females and European males, as well as with the overrepresentation of males among African slaves brought to North America (about 70%) (Davis, 2001; Eltis & Richardson, 2010; Fredrickson, 1981; Painter, 2006) (e.g., see *Trans‐Atlantic Slave Trade Database* website). Notably, these results show that Gullah and JHS African Americans have differing degrees of sex‐biased ancestry contributions, with the Gullah exhibiting a greater male‐biased European contribution (Table 1). These results are consistent with the negligible European female and higher European male contributions previously noted in the Gullah (Parra et al., 2001).

As recently reported (Micheletti et al., 2020), the extent of this sex bias towards European male and African female genetic contributions is known to vary across the Americas due to regional differences in slavery practices. Despite the lack of direct ethnographic records on mating patterns for the Gullah, the evidence that virtually all European X‐chromosomes came from men suggests that few, if any, European descended women produced children in the Gullah community. Plantation owners favored males and children for commodity labor, whereas enslaved female were often situated in more domestic contexts to bear and raise children, while also being sold at greater frequency within the internal slave market in the U.S. (Davis, 1995; Joyner, 1985; Malone, 1992; Rosengarten, Chaplin, & Walker, 1986; Wood, 1974). Yet, given the complex mechanistic models of historical admixture (Goldberg & Rosenberg, 2015), the explanation for our genetic results will require further substantiation by other studies.

### 3.5 African ancestry of Gullah African Americans

We next tried to elucidate whether the genetic data supported a postulated Sierra Leone (Opala, 1987) or diverse African (Pollitzer, 1999) ancestry for the Gullah. Ancestry estimates (Figure 2) suggested that, relative to JHS African Americans, the Gullah had comparable Yoruba ancestry and higher ancestry from the African Rice Coast (from Senegal down to Liberia). As shown in Figure 3, a gradient in the clustering of the Gullah and non‐Gullah African Americans indicated the Gullah's relative proximity to the Sierra Leone (especially Creole) and Mandenka samples, while non‐Gullah African Americans' appeared closer to the Yoruba. A PerMANOVA revealed significant differences (*p* < 0.001) in the principal components for each African American group, and analysis of the individual principal components demonstrated large shifts in PC1 and PC2 (Figure 3). Thus, although not forming individual clusters, the Gullah and non‐Gullah samples were distributed along a gradient in this PCA, with the Gullah samples showing closer Sierra Leone relatedness than the JHS samples.

Close relatives are expected to share large identical‐by‐descent (IBD) segments, which can then be used to model recent ancestry and elucidate population‐level relatedness. Analysis of the mean number of shared IBD segments between pairs of Gullah and JHS African American individuals confirmed that, relative to JHS African Americans, Gullah individuals had a lower mean number of shared European segments and a higher number of shared African segments, including a slightly higher proportion of segments of Mandenka ancestry (Figure S7).

Furthermore, given the relationship between fixation index (*F*_ST_) estimates and admixture levels (Boca & Rosenberg, 2011), we computed the *F*_ST_ (Weir & Cockerham, 1984) to assess the genetic divergence between the admixed groups and their parental source populations. The *F*_ST_ estimates were smaller between the Gullah and the Yoruba and Sierra Leone populations than between the JHS African Americans and these same African populations (Table S3). The closeness to African populations parallels the higher African ancestry of the Gullah relative to the JHS African Americans. For both Gullah and non‐Gullah African Americans, *F*_ST_ estimates for either Sierra Leone or Yoruba were similar, confirming the similar closeness of each African American group to both African populations. Collectively, these data support the closeness of the Gullah to putative ancestral African populations and the interpretation that the Gullah are not direct and exclusive descendants of populations from Sierra Leone. Instead, as postulated by Pollitzer (1999), the Gullah share a common ancestry with numerous populations from Sierra Leone and other regions of West Africa.

These results are consistent with the recently reported genetic ancestries of African Americans from the Southeast U.S. (Micheletti et al., 2020). In this large and representative cohort, Micheletti and colleagues found that Southeast African Americans had the highest African ancestry from Nigeria (26–30%), followed by Coastal West Africa (Sierra Leone and the Windward Coast, [~18%]), West Central Africa (~8%), and Senegambia (~7%). There are obvious discordances with the proportions of Africans that arrived in Charleston through the legal slave trade, who were mostly from West Central Africa (~39%), the Windward Coast and Sierra Leone (~23%), and Senegambia (~20%), and only ~5% from the Bights of Benin and Biafra (Pollitzer, 1999). The apparent overrepresentation of Nigerian ancestry can be explained by the trade of enslaved people from the British Caribbean (Micheletti et al., 2020), and the ethnic composition of Africans imported into the British West Indies indicating source areas in the Gold Coast and the Bights of Benin and Biafra (Pollitzer, 1999). On the other hand, the underrepresentation of Senegambian ancestry can be explained by accounts of early trading and high mortality from this region (Micheletti et al., 2020).

### 3.6 Effects of geographic isolation on Gullah African Americans

Since the Gullah have remained a relatively isolated group over the past few centuries, we sought to determine whether this isolation has affected the genetic structure of their populations. Hallmarks of isolated populations include increased frequencies of recessive disorders, reduced genetic diversity, and higher identity‐by‐descent (IBD) as the result of founder events and population bottlenecks. There are no reports of the increased frequency of any recessive disorders in the Gullah that would support the occurrence of founder events. We first compared the genetic diversity of the Gullah and Sierra Leone populations by measuring mean heterozygosity and inbreeding coefficients. We observed very similar, though slightly lower level of heterozygosity (*p* = 2.72 × 10^−3^) (Table 3 and Table S4) and higher inbreeding coefficient (*p* = 8.78 × 10^−3^) (Table 3 and Table S5, Figure S8), in Gullah compared with Sierra Leone individuals. The similarity in heterozygosity noted in Gullah and Sierra Leone individuals, despite the Gullah's admixture with European individuals, is not unexpected given their low levels of European admixture. This is also the case because the genetic divergence between two West African chromosomes is similar to that between a West African and a European chromosome.

**TABLE 3:** Genetic diversity in Gullah African American and Sierra Leone populations

Population	HETexp	HETobs	*F*
Gullah	0.332	0.333^†^	−0.0018^†^
Sierra Leone	0.332	0.334	−0.0045

*Note*: Expected heterozygosity (HETexp), observed heterozygosity (HETobs), and inbreeding coefficient (*F*) for the Gullah African American and Sierra Leone populations.

^†^ *p* < 0.01 compared with the Sierra Leone population (Wilcoxon test).

**FIGURE 4:**
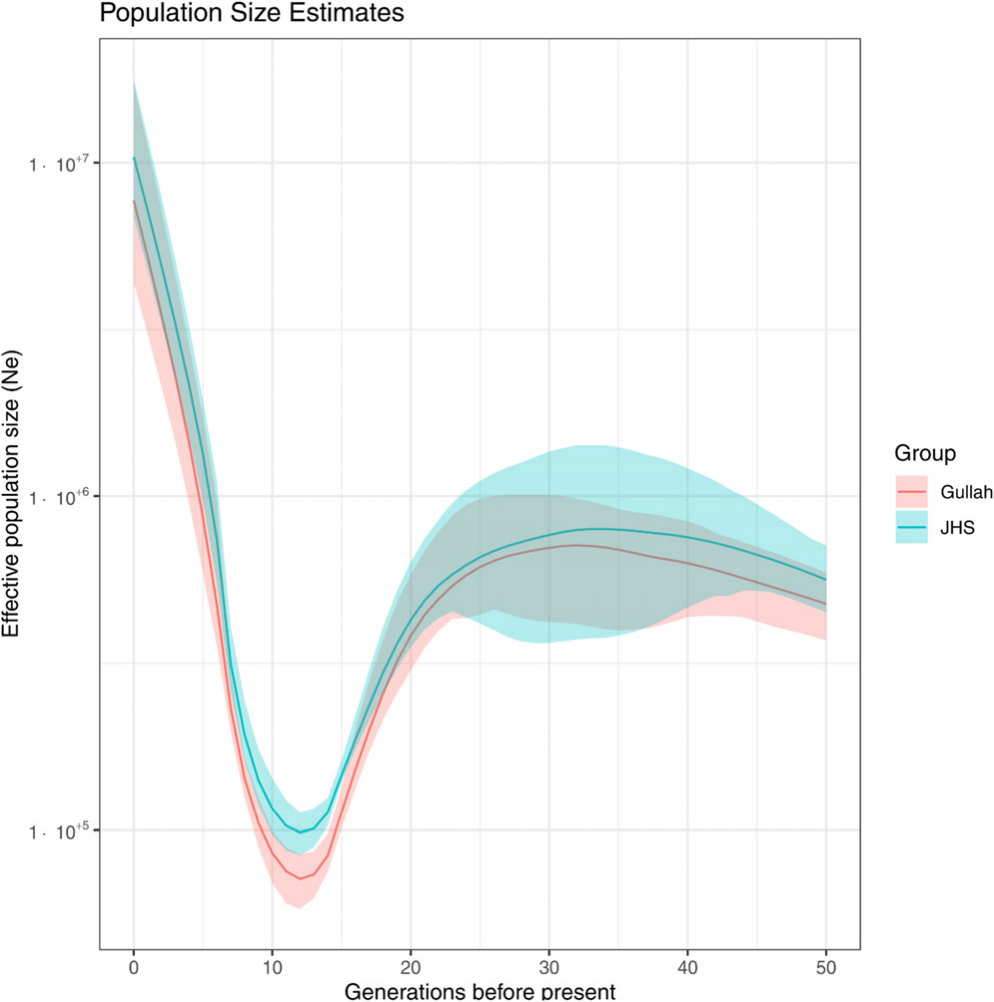
Estimation of ancestry‐specific recent effective population size from segments of identity by descent (IBD) in Gullah and JHS African Americans. Plot displays the recent effective population size (Ne) in Gullah and JHS African Americans over the past 50 generations. The lines show the estimated effective population size based on IBD segments associated only with African ancestry, while the colored regions show 95% bootstrap confidence intervals. The graph displays a bottleneck event occurring nearly 13 generations ago, an estimate consistent with the turn of the 18th century. We used the ancestry‐specific IBDNe pipeline to estimate the effective population sizes

A comparison of the different proportions of IBD segments shared in the Gullah and the JHS African Americans showed a lower mean number of shared European segments and a higher number of shared African segments in the Gullah (Figure S7). This increased number of long founding African haplotypes in the Gullah supports their increased proximity to West African populations, and is consistent with their relative geographic isolation (Pollitzer, 1999). When using IBDNe to explore IBD in African American samples over recent generations (Browning et al., 2018), we noted that the estimated effective population sizes, based only on the African ancestry‐associated IBD segments, in African American groups were mostly similar to each other (Figure 4). This result suggested historical mixing within the larger African ancestry population that encompasses these groups, causing the two Southeast African American groups to have a shared demographic history.

This analysis further revealed a bottleneck event 13 generations ago for both groups (Figure 4), an estimate consistent with the turn of the 18th century. We infer that these bottlenecks mostly resulted from migration and death during the enslavement process. At the same time, these data also support somewhat different demographic histories for the two populations. Based on the African ancestry‐associated IBD segments, Gullah have a slightly tighter bottleneck and lower estimated current effective size than JHS, a finding that is consistent with the higher number of long African segments of IBD among Gullah than JHS (Figure S7). Non‐Gullah African Americans from the Southeast US might therefore have a more diverse geographical origin on average than the Gullah, leading to the larger current estimated effective population size. However, the more apparent bottleneck in the Gullah could also be a bias due to the reduced European admixture in the Gullah. Collectively, these results are consistent with the relative isolation of the Gullah.

## 4 CONCLUSION

Our study helps clarify the debated ancestry of Gullah African Americans and makes clear that Sierra Leone is not the sole origin point. We confirmed their higher African, lower European, and small Native American ancestries, as well as a larger proportion of male‐biased European admixture. We show that the Gullah have a diverse African ancestry, with increased proximity to West African populations than Southeastern non‐Gullah African Americans. We found genomic evidence for a slightly tighter bottleneck in the Gullah consistent with a founder event(s) upon importation to the U.S. These findings are consistent with historical, cultural, and anthropological evidence indicating that their relative geographical isolation and strong community life allowed the Gullah to preserve many aspects of their African cultural heritage (Pollitzer, 1999). Although the subtle genetic differences relative to Southeastern non‐Gullah African Americans support somewhat different demographic histories, these results also reveal largely shared common ancestries. As such, our data shows that the Gullah are not a genetically distinct group per se, but rather a culturally distinct group of African Americans with subtle variation in its genetic structure.

Broadly, this study reveals subtle differences in genetic structure and ancestry in African American populations. These differences can have important implications for precision medicine, and further reveal the crucial need to include more ancestrally diverse individuals in medical genomic studies (Dai et al., 2020). Only a comprehensive understanding of the genetic architecture of these populations can ensure that they are not omitted from developments in new genetic technologies and clinical advancements, ultimately contributing to the closure of the health disparities gap as healthcare moves towards precision medicine. Finally, this study is important for Gullah and non‐Gullah African Americans who were stripped of ancestral identities by the slave trade. Combined with socio‐historical resources, this research can help to recover ancestral histories, and contribute to their new collective identities and ties to ancestral homelands, ultimately paving the road towards transforming lives and possibly reconciliation (Nelson, 2016).

## CONFLICT OF INTEREST

The authors declare no conflicts of interest.

## AUTHOR CONTRIBUTIONS

**Kip Zimmerman:** Data curation; formal analysis; investigation; validation; visualization; writing‐original draft; writing‐review & editing. **Theodore Schurr:** Investigation; writing‐original draft; writing‐review & editing. **Wei‐Min Chen:** Conceptualization; data curation; investigation; methodology; writing‐original draft; writing‐review & editing. **Uma Nayak:** Data curation; investigation; writing‐original draft; writing‐review & editing. **Josyf Mychaleckyj:** Investigation; writing‐original draft; writing‐review & editing. **Queen Quet:** Investigation; writing‐original draft; writing‐review & editing. **Lee Moultrie:** Investigation; writing‐original draft; writing‐review & editing. **Jasmin Divers:** Data curation; investigation; writing‐original draft; writing‐review & editing. **Keith Keene:** Investigation; writing‐original draft; writing‐review & editing. **Diane Kamen:** Funding acquisition; resources; writing‐original draft; writing‐review & editing. **Gary Gilkeson:** Funding acquisition; resources; writing‐original draft; writing‐review & editing. **Kelly Hunt:** Investigation; resources; writing‐original draft; writing‐review & editing. **Ida Spruill:** Investigation; resources; writing‐original draft; writing‐review & editing. **Jyotika Fernandes:** Funding acquisition; resources; writing‐original draft; writing‐review & editing. **Melinda Aldrich:** Investigation; validation; writing‐original draft; writing‐review & editing. **David Reich:** Conceptualization; formal analysis; investigation; methodology; writing‐original draft; writing‐review & editing. **W. Timothy Garvey:** Conceptualization; funding acquisition; investigation; resources; validation; writing‐original draft; writing‐review & editing. **Carl Langefeld:** Conceptualization; formal analysis; investigation; methodology; validation; writing‐original draft; writing‐review & editing. **Michele Sale:** Conceptualization; funding acquisition; investigation; project administration; resources; supervision; writing‐original draft; writing‐review & editing. **Paula Ramos:** Conceptualization; funding acquisition; investigation; project administration; supervision; validation; writing‐original draft; writing‐review & editing.

## Supporting information

**Appendix S1**. Supporting Information

## Data Availability

All genotypic data used in this study have been deposited in public databases. Gullah African American and African (Sierra Leone) data can be accessed through dbGAP with the accession code phs000433.v1.p1 (The Sea Islands Genetic Network (SIGNET)).
