# Supplementary material for: Genetic landscape of Gullah African Americans
Source: Am J Phys Anthropol. 2021 May 19;175(4):905–19. doi: 10.1002/ajpa.24333 (PMC8286328; doi:10.1002/ajpa.24333)
Supplement: Supplementary file 1 — Appendix S1. Supporting Information [file AJPA-175-905-s001.docx]

**Supporting Information for**

Genetic Landscape of Gullah African Americans

Kip D. Zimmerman, Theodore G. Schurr, Wei-Min Chen, Uma Nayak, Josyf C. Mychaleckyj, Queen Quet, Lee H. Moultrie, Jasmin Divers, Keith L. Keene, Diane L. Kamen, Gary S. Gilkeson, Kelly J. Hunt, Ida J. Spruill, Jyotika K. Fernandes, Melinda C. Aldrich, David Reich, W. Timothy Garvey, Carl D. Langefeld, Michèle M. Sale, Paula S. Ramos*

Corresponding author: Paula S. Ramos

Email: [ramosp@musc.edu](mailto:ramosp@musc.edu)

**This PDF file includes:**

Figures S1 to S8

Tables S1 to S5

References for Supporting Information reference citations

| 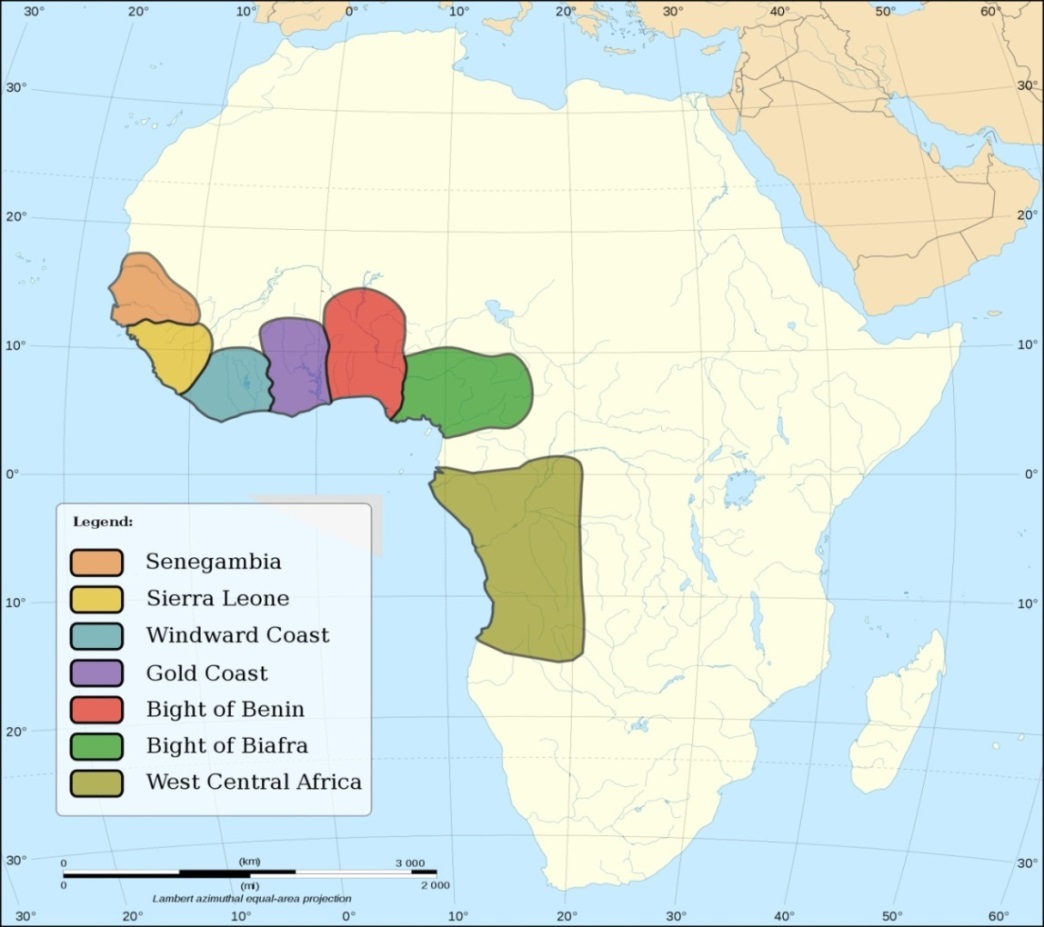 |
| --- |
| **Figure S1.** **Major slave exportation regions in Africa during 15th–19th centuries.** Senegambia (Gambia and Senegal), Sierra Leone (Guinea and Sierra Leone), Windward Coast (Ivory Coast and Liberia), Gold Coast (Ghana), Bight of Benin (from the Volta River to the Benin River), Bight of Biafra (east of the Benin River to Gabon), and Angola (west central Africa, including part of Gabon, Congo, and Angola). (Modified by Grin 20 based on the original [Africa_map_blank.svg](http://commons.wikimedia.org/wiki/File:Africa_map_blank.svg) by Eric Gaba under the Creative Commons Attribution-Share Alike 2.5 Generic, 2.0 Generic and 1.0 Generic license and the [Creative Commons](http://en.wikipedia.org/wiki/en:Creative_Commons) [Attribution-Share Alike 3.0 Unported](http://creativecommons.org/licenses/by-sa/3.0/deed.en) license. [*http://en.wikipedia.org/wiki/File:Africa_slave_Regions.svg*](http://en.wikipedia.org/wiki/File:Africa_slave_Regions.svg)*)* |

| 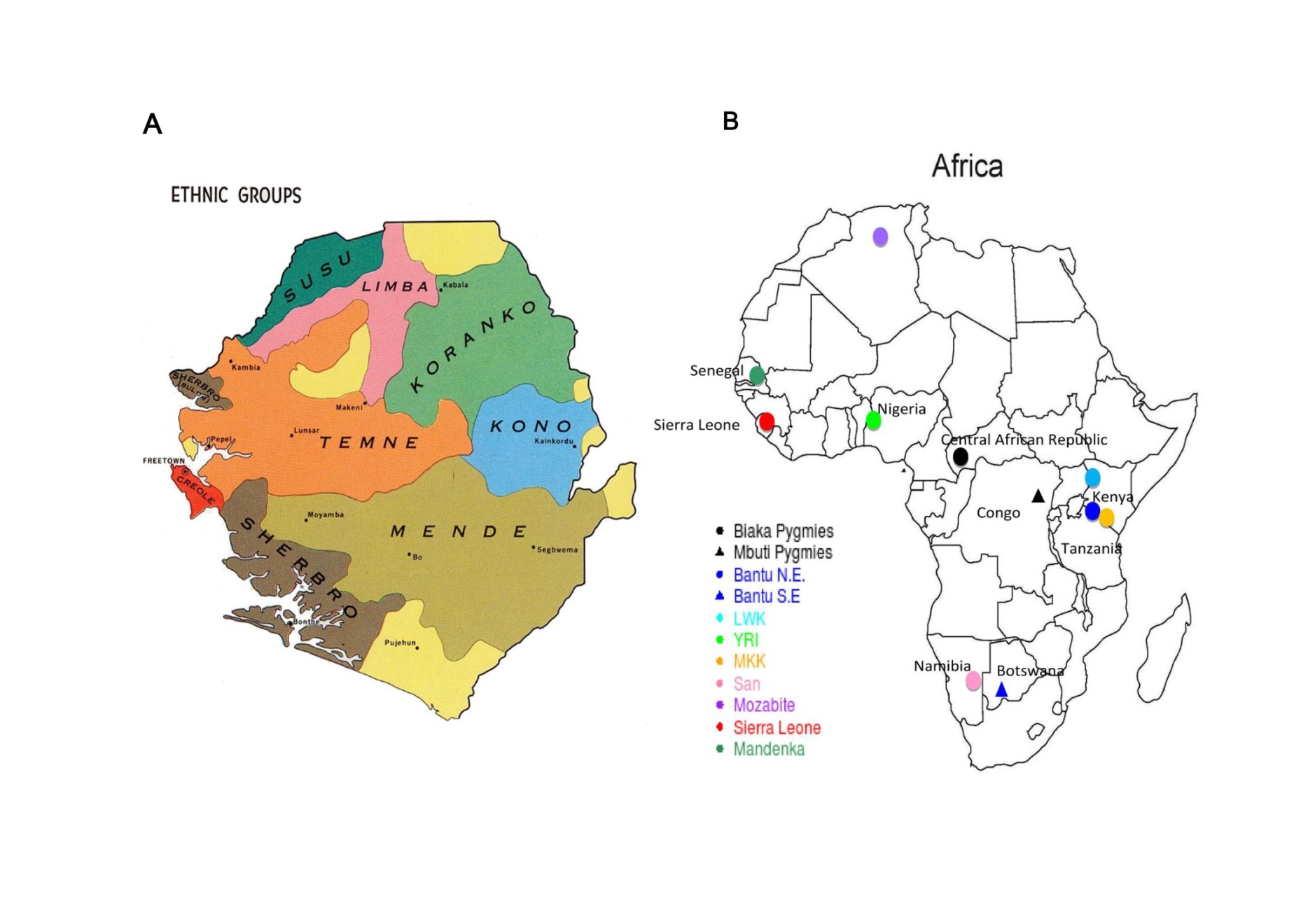 |
| --- |
| **Figure S2.** **Sierra Leonean ethnic groups.** Map of Sierra Leone and its ethnic groups groups. Our data set has samples from 10 ethnic groups of Sierra Leone (Creole, Fullah, Kono, Kroo, Limba, Loko, Madingo, Mende, Sherbro, Susu, Temne) (Figure courtesy of the University of Texas Libraries, the University of Texas at Austin). |

| 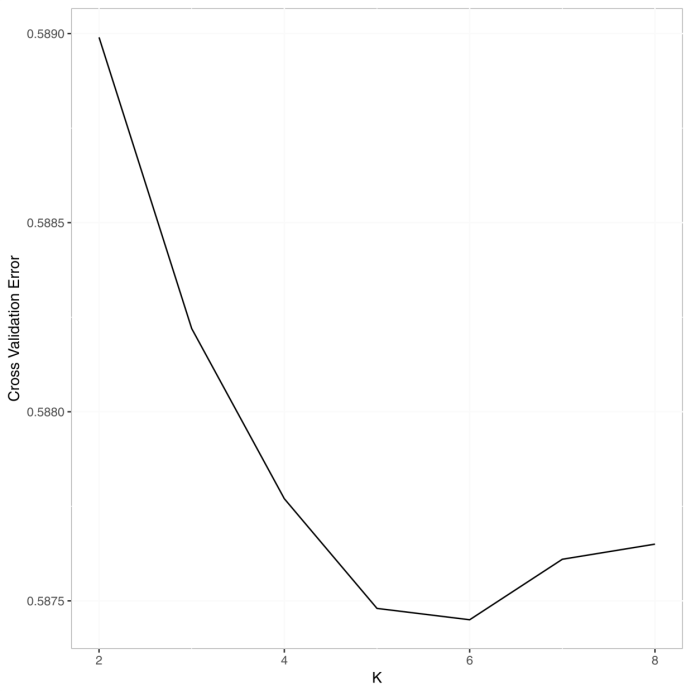 |
| --- |
| **Figure S3. Cross-validation (CV) error plot in the ADMIXTURE analysis to determine optimal *k* for all subjects.** The most appropriate *k* was 5. |

| 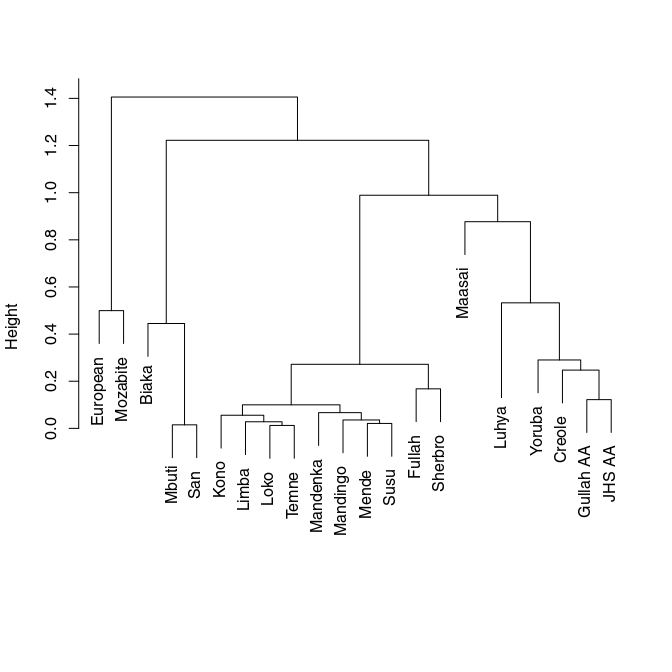 |
| --- |
| **Figure S4. Dendrogram used to order populations for the ADMIXTURE plot (shown in Figure 2).** Populations were clustered via the means of each the five ancestral populations (*k*). Using the five values for each population, a hierarchical cluster analysis was computed using an inter-population similarity matrix of Euclidean distances. Using the similarity matrix, each population begins as its own cluster and the algorithm proceeds to iteratively join the two most similar clusters until all clusters are joined. AA: African American; JHS: Jackson Heart Study African Americans from Jackson, Mississippi. |


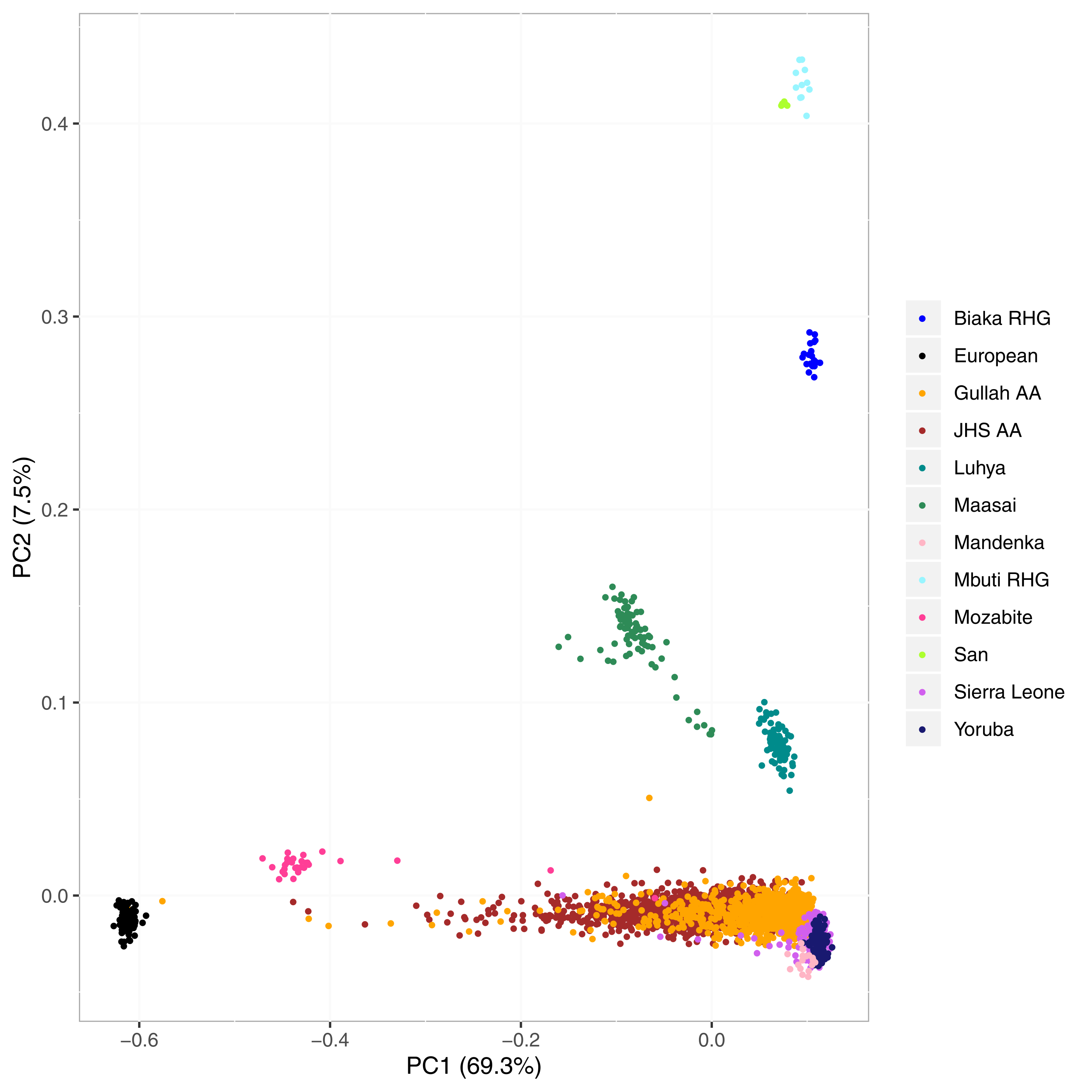


Figure S5. Principal component analysis of all African and African American samples. Principal component analysis (PCA) (EIGENSOFT) was applied to HGDP and HapMap III African, African American, and Sierra Leone populations. PCA shows the relative similarity of the African American populations compared to the Sierra Leone, Yoruba, and Mandenka populations.

| **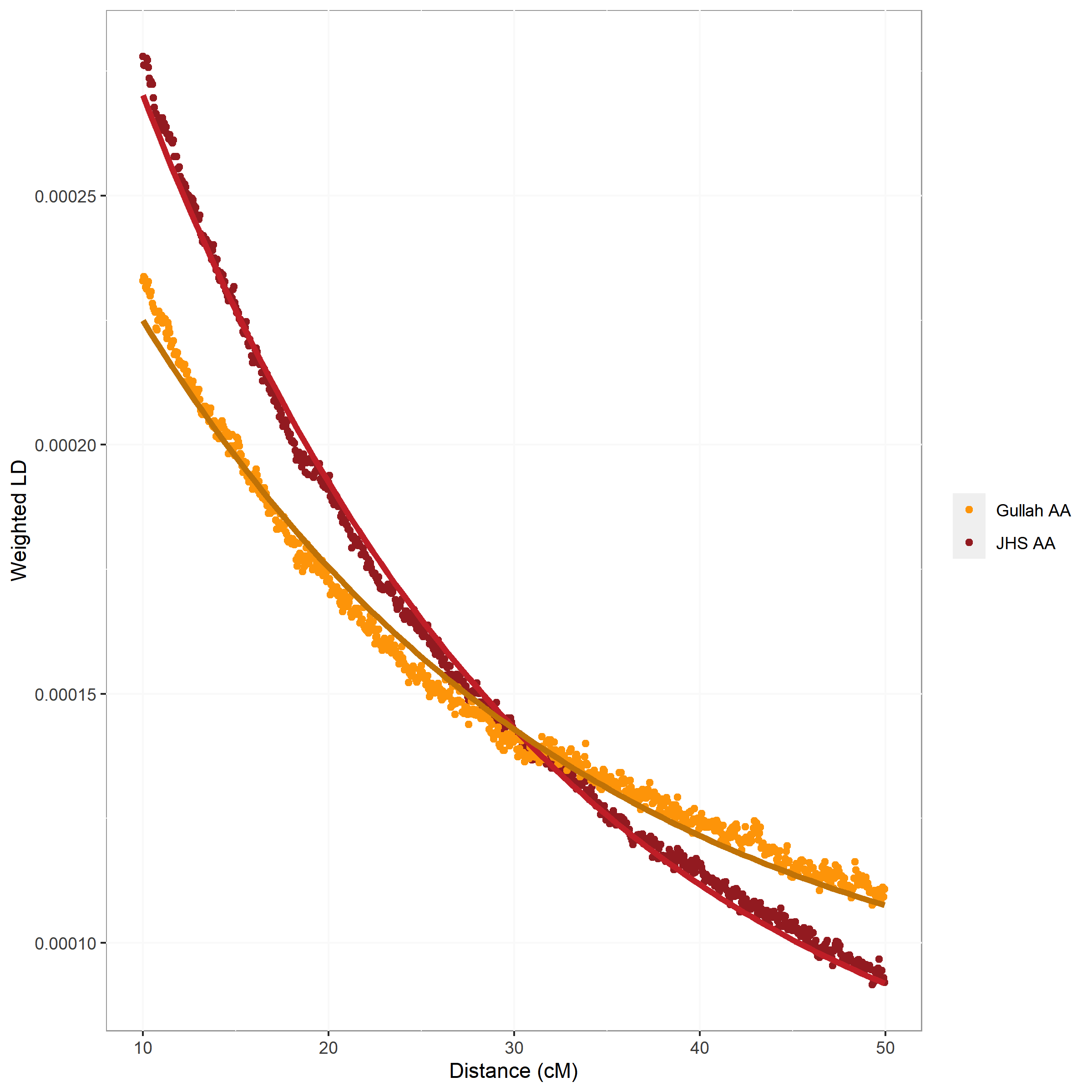** |
| --- |
| **Figure S6. Weighted linkage disequilibrium (LD) decay curves for Gullah and JHS African Americans using CEU and YRI as sources.** |

| 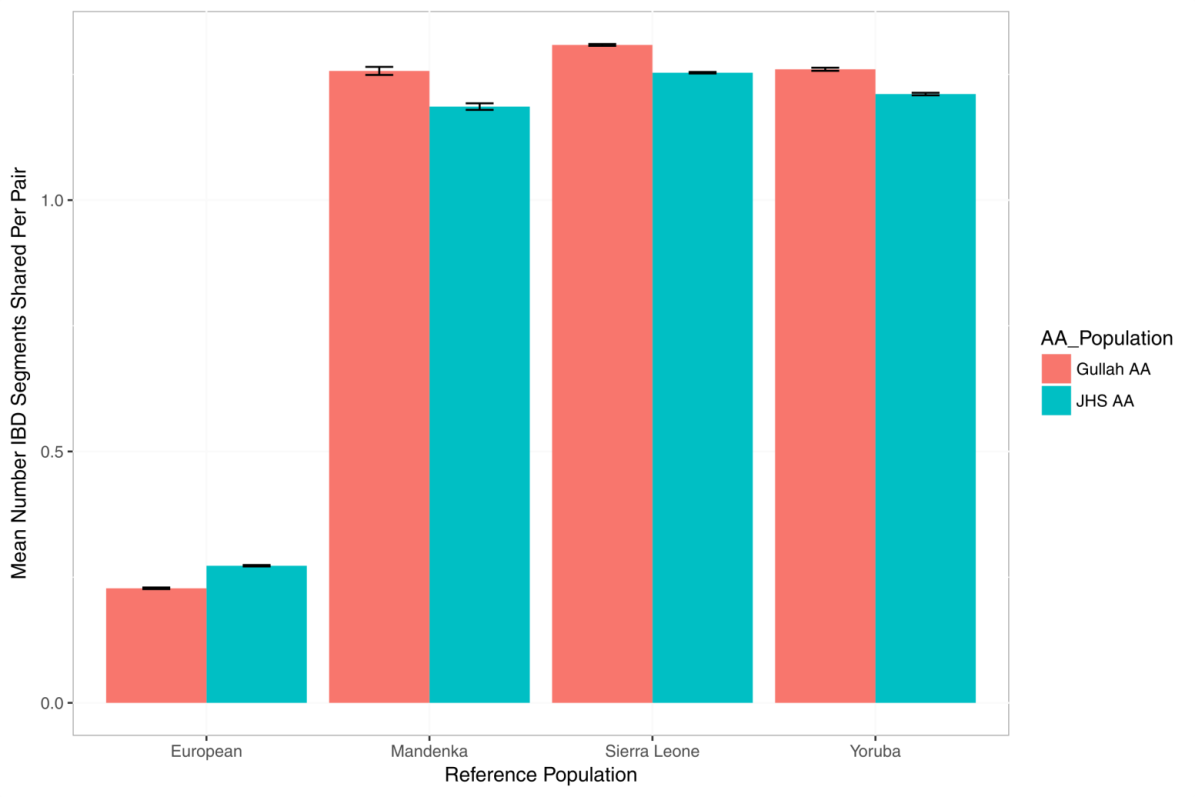 |
| --- |
| **Figure S7. Identity by Descent (IBD) in Gullah and JHS African Americans reflects their ancestry.** Histogram shows mean number of shared segments of IBD between pairs of individuals. We used GERMLINE to compute the sharing of long IBD segments (*l* ≥ 18cM), which are indicative of recent relatedness. Relative to the JHS, the Gullah African Americans show less European and more African IBD segments. Also, compared to JHS, the Gullah show a slightly higher proportion of segments with the Mandenka. |

| 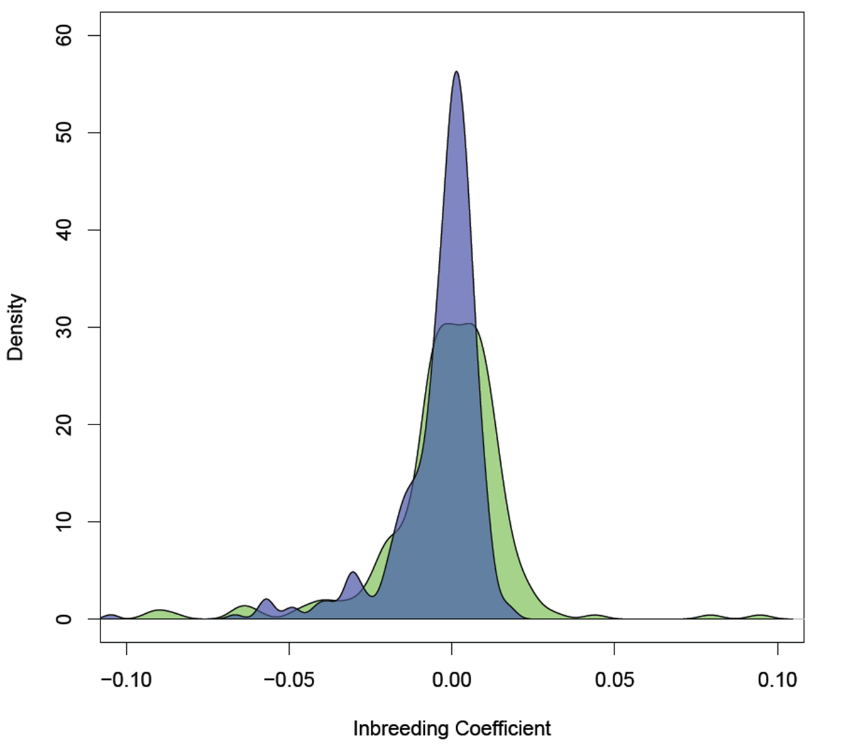 |
| --- |
| **Figure S8. Density plot of the inbreeding coefficients, *F*, for both the Gullah African American (green) and Sierra Leone (purple) populations.** |

| **Table S1: African American, Native American, and African Samples and Variants by Study after Quality Control** | | | |
| --- | --- | --- | --- |
| **Study** | **N** | **Variants** | **Array** |
| **Gullah AA** | 883 | 699,356 | Affymetrix 6.0 |
| **JHS AA (non-Gullah AA)** | 1,322 | 749,518 | Affymetrix 6.0 |
| **Sierra Leone Africans** | 381 | 699,356 |  |
| Mende | 204 |  |  |
| Temne | 90 |  |  |
| Creole | 33 |  |  |
| Limba | 15 |  |  |
| Susu | 10 |  | Affymetrix 6.0 |
| Mandingo | 10 |  |  |
| Loko | 8 |  |  |
| Fullah | 6 |  |  |
| Sherbro | 4 |  |  |
| Kono | 1 |  |  |
| **HGDP** | 125 | 572,940 | Illumina HumanHap650Y |
| Mozabite | 29 |  |  |
| Biaka RHG | 22 |  |  |
| Yoruba | 21 |  |  |
| Mandenka | 19 |  |  |
| Mbuti RHG | 11 |  |  |
| San | 6 |  |  |
| **HapMap III** | 386 | 1,201,862 | Affymetrix 6.0 and Illumina 1M |
| European | 112 |  |  |
| Yoruba | 111 |  |  |
| Luhya | 80 |  |  |
| Maasai | 83 |  |  |
| **Native Americans** |  |  | Illumina Human660W |
| Mixtec | 7 | 432,378 |  |
| **Merged & Filtered** | 3,087 | 136,878 |  |
| **Merged, Filtered, & LD Pruned** | 3,087 | 64,303 |  |
| JHS AA: African Americans (AA) from the Jackson Heart Study (JHS); RHG: rainforest hunter-gatherers. | | | |

| **Table S2.** **Geographic and linguistic distribution of the African populations used in this study.** | | | |
| --- | --- | --- | --- |
| **Population** | **Region** | **Country** | **Language family** |
| Mozabite | Northern Africa | Algeria | Afroasiatic |
| Yoruba (YRI) | Western Africa | Nigeria | Niger-Kordofanian |
| Mandenka | Western Africa | Senegal | Niger-Kordofanian |
| Sierra Leonean | Western Africa | Sierra Leone | Niger-Kordofanian |
| Biaka RHG | Middle Africa | Central African Republic | Niger-Kordofanian |
| Mbuti RHG | Middle Africa | Democratic Republic of the Congo | Nilo-Saharan |
| Luhya (LWK) | Eastern Africa | Kenya | Niger-Kordofanian |
| Maasai (MKK) | Eastern Africa | Kenya | Nilo-Saharan |
| San | Southern Africa | Namibia | Khoisan |

RHG: rainforest hunter-gatherers. Note: Regions are defined based on the UN Statistics Division geoscheme (<https://unstats.un.org/> unsd/methodology/m49).

**Table S3. Pairwise F_ST_ estimated between populations**.

|  | **Sierra Leone** | **YRI** | **CEU** |
| --- | --- | --- | --- |
| **Gullah African American** | 0.003 | 0.004 | 0.099 |
| **Non-Gullah African American** | 0.038 | 0.038 | 0.109 |

Non-Gullah are African Americans from the Jackson Heart Study.

YRI = Yoruba, and CEU = CEPH European-Americans.

**Table S4. Expected heterozygosity (HETexp) and observed heterozygosity (HETobs) for the Gullah African American and Sierra Leone populations.**

| **Population** | **HETexp** | | | **HETobs** | | | **Wilcoxon Test P-value** |
| --- | --- | --- | --- | --- | --- | --- | --- |
|  | **Mean** | **Median** | **SD** | **Mean** | **Median** | **SD** |  |
| **Gullah** | 0.332 | 0.332 | ±0.00017 | 0.333 | 0.332 | ±0.0064 | 2.73E-03 |
| **Sierra Leone** | 0.332 | 0.332 | ±0.00019 | 0.334 | 0.332 | ±0.0049 |  |

SD: standard deviation.

| **Table S5. Inbreeding coefficient (*F*) for the Gullah African American and Sierra Leone populations** | | | | |
| --- | --- | --- | --- | --- |
| **Population** | ***F*** | | | **Wilcoxon Test P-value** |
|  | **Mean** | **Median** | **SD** |  |
| **Gullah** | -0.0018 | 0.0005 | ± 0.019 | 8.78E-03 |
| **Sierra Leone** | -0.0045 | -0.0004 | ± 0.015 |  |

SD: standard deviation.
